# Age, sex and angiographic type-related phenotypic differences in inpatients with Takayasu arteritis: A 13-year retrospective study at a national referral center in China

**DOI:** 10.3389/fcvm.2023.1099144

**Published:** 2023-03-16

**Authors:** Jingya Zhou, Jing Li, Yi Wang, Yunjiao Yang, Jiuliang Zhao, Mengtao Li, Haiyu Pang, Tingyu Wang, Yuexin Chen, Xinping Tian, Xiaofeng Zeng, Yuehong Zheng

**Affiliations:** ^1^ Department of Medical Records, Peking Union Medical College Hospital, Chinese Academy of Medical Sciences and Peking Union Medical College, Beijing, China; ^2^ WHO Family of International Classifications Collaborating Center of China, Beijing, China; ^3^ Department of Rheumatology and Clinical Immunology, Peking Union Medical College Hospital, Chinese Academy of Medical Sciences & Peking Union Medical College; National Clinical Research Center for Dermatologic and Immunologic Diseases (NCRC-DID), Ministry of Science & Technology; State Key Laboratory of Complex Severe and Rare Diseases, Peking Union Medical College Hospital (PUMCH); Key Laboratory of Rheumatology and Clinical Immunology, Ministry of Education, Beijing, China; ^4^ Medical Research Center, State Key Laboratory of Complex Severe and Rare Diseases, Peking Union Medical College Hospital, Chinese Academy of Medical Sciences & Peking Union Medical College, Beijing, China; ^5^ Clinical Epidemiology Unit, International Epidemiology Network, Peking Union Medical College Hospital, Chinese Academy of Medical Sciences and Peking Union Medical College, Beijing, China; ^6^ Department of Vascular Surgery, Peking Union Medical College Hospital, Chinese Academy of Medical Sciences and Peking Union Medical College, Beijing, China

**Keywords:** takayasu arteritis, gender, age at onset, angiographic classification, complication

## Abstract

**Backgrounds:**

We aimed to investigate the demographic characteristics, vascular involvement, angiographic patterns, complications, and associations of these variables in a large sample of TAK patients at a national referral center in China.

**Methods:**

The medical records of TAK patients discharged from 2008 to 2020 were retrieved from the hospital discharge database using ICD-10 codes. Demographic data, vascular lesions, Numano classifications and complications were collected and analyzed.

**Results:**

The median age at onset was 25 years in 852 TAK patients (670 female, 182 male). Compared with the females, the male patients were more likely to have type IV and were more likely to have iliac (24.7% vs. 10.0%) and renal artery (62.7% vs. 53.9%) involvement. They also had a higher prevalence of systemic hypertension (62.1% vs. 42.4%), renal dysfunction (12.6% vs. 7.8%) and aortic aneurysm (AA) (8.2% vs. 3.6%). The childhood-onset group was more likely to have involvement of the abdominal aorta (68.4% vs. 52.1%), renal artery (69.0% vs. 51.8%) and superior mesenteric artery (41.5% vs. 28.5%), and they were more likely to have type IV, V and hypertension than the adult-onset group. After adjusting for sex and age at onset, the patients with type II were associated with an increased risk of cardiac dysfunction (II vs. I: OR = 5.42; II vs. IV: OR = 2.63) and pulmonary hypertension (II vs. I: OR = 4.78; II vs. IV: OR = 3.95) compared with those with types I and IV. Valvular abnormalities (61.0%) were observed to be most prevalent in patients with type IIa. The patients with Type III were associated with a higher risk of aortic aneurysm (23.3%) than the patients with types IV (OR = 11.00) and V (OR = 5.98). The patients with types III and IV were more commonly complicated with systemic hypertension than the patients with types I, II and V. *P *< 0.05 in all of the above comparisons.

**Conclusion:**

Sex, adult/childhood presentation and Numano angiographic type were significantly associated with differences in phenotypic manifestations, especially cardiopulmonary abnormalities, systemic hypertension, renal dysfunction and aortic aneurysm.

## 1. Introduction

Takayasu arteritis (TAK), also formerly known as “pulseless disease”, is a rare chronic systemic inflammatory disease involving the aorta and its primary branches. It usually causes granulomatous inflammation of the adventitia and the media of the aorta. The coronary and pulmonary arteries can also be affected. Several studies have shown that there are obvious regional or ethnic differences in the epidemiological and clinical features of TAK. The incidence of TAK in East Asia, South Asia and Latin America is reported to be much higher than that in Europe and the United States (1). In terms of its clinical features, Japanese TAK patients suffer more from aortic insufficiency and show higher involvement of the aortic arch and its branches (2), while Indian patients often show symptoms of hypertension and tend to have more frequent vascular involvement of the abdominal aorta and its branches (3).

Previous studies have also suggested that the demographic characteristics may affect the clinical features of TAK patients. It has been reported that male TAK patients are more likely to have abdominal artery involvement (2, 4, 5), hypertension (3, 4) and aortic aneurysm (AA) (6, 7). A study from Japan demonstrated that abdominal vascular lesions are more prevalent in male TAK patients with an age at onset over 40 years (2). Moreover, aortic and renal artery involvement were more frequently observed in pediatric TAK patients (8). The Chinese TAK cohorts have been limited because the published studies have only investigated the association between demographic characteristics and vascular involvement (9) or have investigated the clinical features with a specific complication (7, 10–19). To our knowledge, there are still gaps in understanding the association between the angiographic classifications and phenotypic manifestations. This study aims to investigate the demographic characteristics, vascular lesions, angiographic classifications, complications and the association among these factors in a large cohort of TAK inpatients.

## 2. Methods

This is a retrospective study conducted at Peking Union Medical College Hospital, which is a national referral center proficient in diagnosing and managing TAK with multidisciplinary team collaboration (9, 16–20) in China. This study was carried out in accordance with the tenets of the Helsinki Declaration and was approved by the Review Board of PUMCH (S-K2122).

### 2.1. Data collection of patients

**Figure 1:**
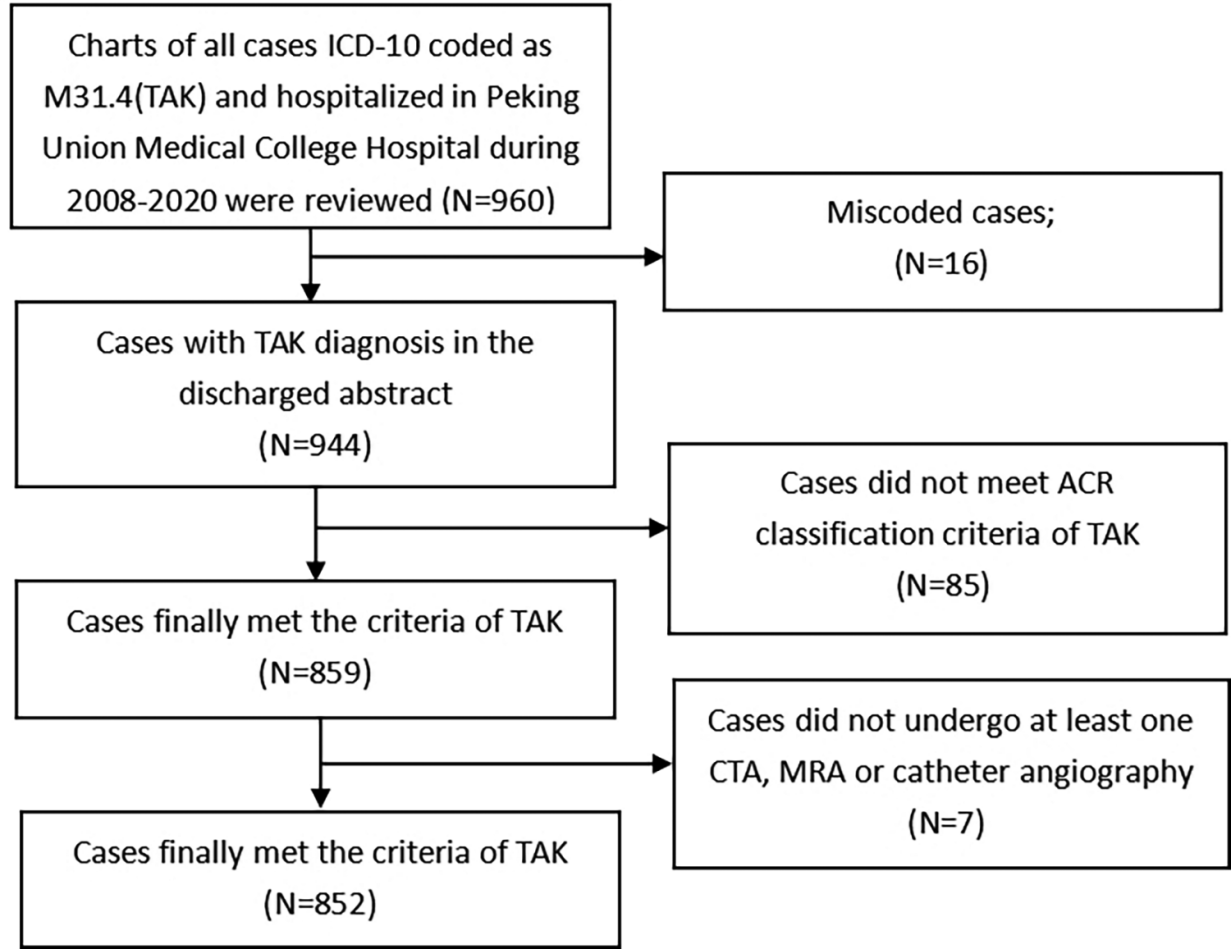
The finding strategy for recruiting the TAK patients in this study. Legend: This figure indicates the flow chart of the patients included in our study.

Since diagnoses on discharge are routinely assigned ICD-10 codes by clinical coders, we retrieved patients with TAK who were admitted from January 1, 2008, to December 31, 2020, by searching for the ICD-10 code M31.4 within the hospital discharge database that is managed by the department of medical records. To minimize information bias caused by coding errors, all the TAK patients' original discharge abstracts were further reviewed by the authors to ensure that the patient met the 1990 ACR diagnostic criteria of TAK (21). Eighty-five patients who did not meet the criteria, 16 with the wrong codes were excluded from our study. Seven patients who did not undergo at least one computed tomography angiography (CTA), magnetic resonance angiography (MRA) and catheter angiography were also excluded from our study. The patient-finding strategy is shown in Figure 1.

The demographic data, diagnosis on discharge with corresponding ICD-10 codes, surgical procedures and the ICD-9-CM-3 codes for each of these patients were extracted directly from the hospital discharge database. Detailed information about age at onset and vascular lesion distribution were further extracted from the original medical records. If the same patient had multiple hospitalizations during the study period, then all the extracted data for each admission were merged into one record. According to the age at onset, we divided all the patients into two groups: the childhood-onset patients with an age at onset less than 18 years old and the adult-onset patients with an age at onset greater than or equal to 18 years old.

For each patient, the findings from vessel imaging examinations by catheter angiography, MRA and CTA were evaluated. For the patients who had possible intracranial, pulmonary, or coronary vessel involvement, the vessel imaging results shown by CTA or MRA were also assessed. The typical lesions of arteries included stenosis, occlusion, dilation or aneurysm and artery wall thickening detected by the above examinations was recorded as vascular lesion. According to the vascular involvement distribution, each patient was grouped based on the Numano classification proposed in 1994 (22). Given that the Numano angiographic types were central to the data analysis in this study, two radiologists were invited to review the angiographic images independently and determine the Numano angiographic types in each patient. When a patient underwent multiple vessel imaging examinations, the study with the most extensive involvement of vascular lesions was recorded for further analysis.

Because the complications of TAK patients are routinely diagnosed by clinicians according to the specific diagnostic criteria at the time, after referencing the literature (23, 24), several of the most concerning complications of TAK patients were identified and retrieved by the ICD-10 codes for each patient in the hospital discharge database, including hypertension (I10–I15, H35.0 and I67.4), tuberculosis (A16–A19, B90.9), infectious diseases of the respiratory system (J00–J22, J85–J86, J44.0–J44.1), pulmonary hypertension (I27.0, I27.2), ischemic heart disease (I20–I25), cardiac dysfunction (I50), nonrheumatic heart valve disease (I34–I38), ischemic stroke (I60–I64/I69), transient cerebral ischemia (G45), renal dysfunction (N17–N19, I12.0), contracted kidney (N26), and aortic aneurysm (I71.1–I71.9). Within the patient group with an ICD-10 code of ischemic heart disease, we further excluded those patients with coronary atherosclerotic heart disease (mainly dependent on image findings of coronary arteries combined with opinion of senior physicians from departments of rheumatology and cardiology).We reviewed the original medical records to further verify those complications, making sure that they met the corresponding diagnostic criteria at discharge. In our study, pulmonary hypertension was defined when estimated pulmonary arterial systolic pressure ≥40 mmHg and peak systolic velocity of the tricuspid valve >3.4 m/s upon echocardiography (25) or mean pulmonary arterial pressure ≥25 mm Hg at rest by right heart catheterization was found (26). The complications that occurred at the first admission or during the follow-up were included in the comparison analysis for the groups of different Numano types.

### 2.2. Statistical analysis

SPSS for Windows, version 19.0 (SPSS Inc., IBM, United States) was used to analyze the data. Continuous data were described as the median (quartile1 quartile3). Categorical data are expressed as frequency (percentage). The medians were compared using the Wilcoxon rank-sum test. The percentages were compared using the chi-square test. Multivariate logistic regression analyses were applied to evaluate the association between the Numano angiographic pattern and the prevalence of each complication. In this model, we set complications as the dependent variable (1 = present, 0 = absent) and the Numano angiographic pattern, age at onset and sex were set as covariates. An adjusted odds ratio (OR) with 95% CI was calculated. Statistical significance was defined as a two-tailed *P*-value <0.05.

## 3. Results

### 3.1. Demographic data and Numano's angiographic classifications

**Figure 2:**
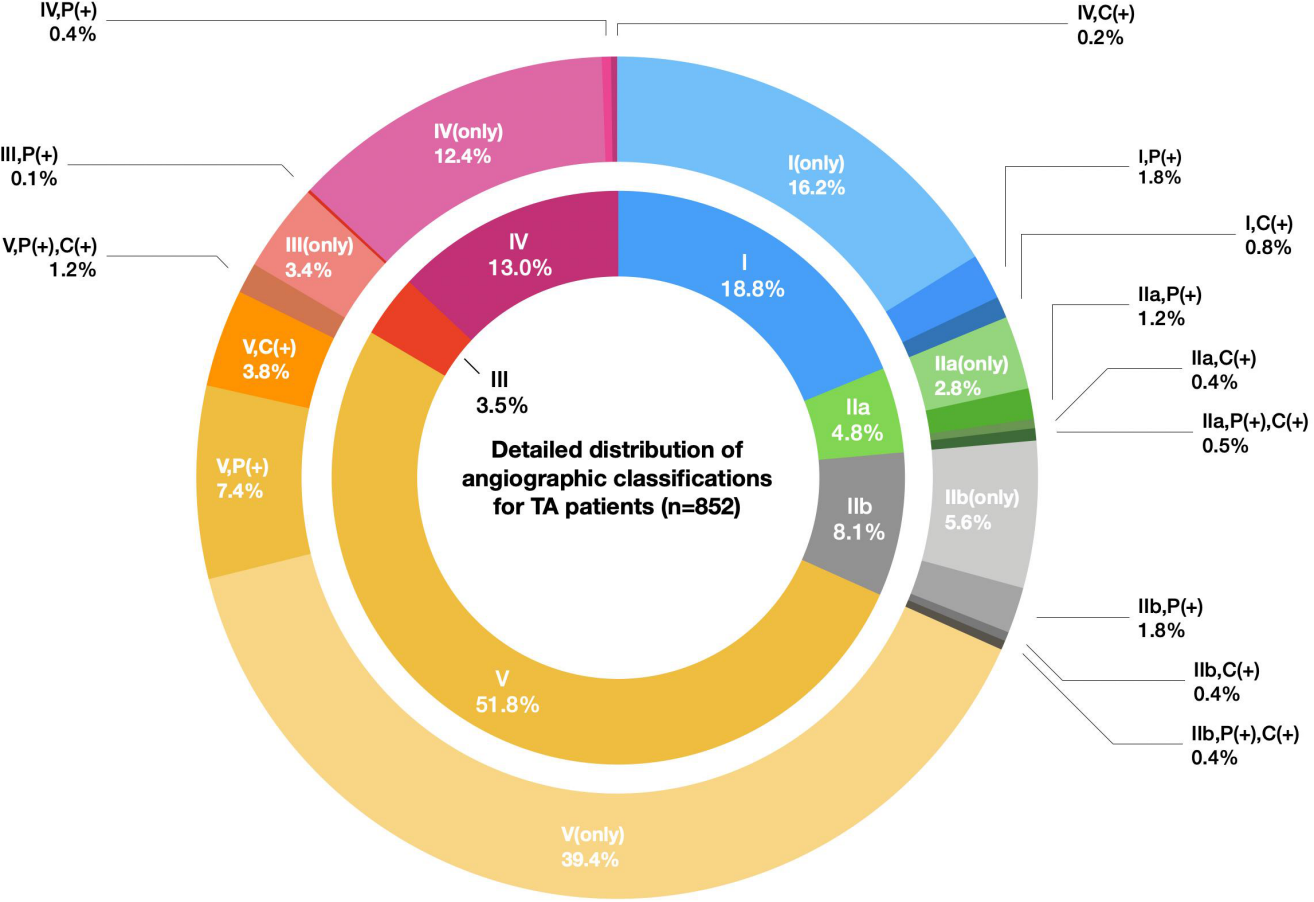
Detailed distribution of the angiographic classifications for TA patients (*n* = 852). Legend: The inner circle indicates the proportions of six Numano angiographic patterns I–V, and the outer circle represents the detailed proportions of the Numano angiographic patterns I–V plus the involvement of the pulmonary artery or coronary artery. The fragments with the same color scheme in the inner and outer circles belong to a specific type of Numano angiographic pattern I–V.

A total of 852 TAK patients were finally recruited (670 female, 182 male), and the F:M ratio was 3.7:1. The median age (Q1,Q3) at onset was 25 (18, 33) years for all TAK patients. The age at onset displayed no sex difference, with a median age at onset of 25 (17, 33) for males and 25 (19, 32) for females (*P *= 0.347). The female patients had a peak age of onset at 20–24 years, while males had a bimodal peak age of onset at 15–19 years and 25–29 years. (Supplementary Figure S1). In addition, we did not observe a different F:M ratio between childhood-onset and adult-onset groups (2.89:1 vs. 3.96:1, *P *= 0.107). A total of 32.5% of the patients underwent surgical treatment or endovascular intervention at different stages of disease, while 67.5% of the patients received medical treatment only. Numano type V (51.8%) was the most common angiographic pattern in TAK patients, followed by type I (18.8%) and type IV (13.0%). A more detailed classification of the angiographic patterns is summarized in Figure 2.

### 3.2. Distribution of the vascular lesions and comparison between the sexes and age at onset

All the patients underwent at least one CTA, MRA and conventional angiography (803 CTA, 139 MRA and 288 conventional angiography). Among them, 129 patients were evaluated by both CTA and MRA, 176 by both CTA and conventional angiography and 37 by both MRA and conventional angiography. Angiographic findings showed that the carotid artery (76.0.%) and subclavian artery (73.7%) were the most commonly involved arteries, followed by the abdominal aorta (56.0%) and renal artery (55.8%). Besides, the percent of patients with involvement of the pulmonary arteries and coronary arteries were 68.5% and 38.0% in those patients who received corresponding catheter angiography or CTA, respectively. The iliac artery (24.7% vs. 10.0%, *P *< 0.001) and renal artery (62.7% vs. 53.9%, *P *< 0.046) were more likely to be affected in the male patients than in the female patients. Conversely, the innominate artery, carotid artery and subclavian artery were found to be more frequently affected in the female patients than in the male patients (46.0% vs. 30.6%, *P < *0.001; 78.2% vs. 67.5%, *P *= 0.003; 78.0% vs. 56.9%, *P < *0.001). The childhood-onset group were more likely to have involvement of the abdominal aorta, renal artery and superior mesenteric artery (*P* < 0.001 for all comparisons). In the adult-onset group, the ascending aorta, aortic arch, innominate artery and carotid artery were more likely to be involved than in the childhood-onset group (*P *< 0.05 for all comparisons). The overall detailed vascular involvement among the sex and different age groups of onset is summarized in Table 1.

**Table 1:** Distribution of the arteries involved in TAK patients among the gender and age groups of onset [*n*(%)].

		Sex			Age at onset (year)		
Vascular site	Total (*n* = 852)	Male (*n* = 182)	Female (*n* = 670)	*P*	<18 (*n* = 183)	≥18 (*n* = 669)	*P*
Ascending aorta	179/728 (24.6)	42/149 (28.2)	137/579 (23.7)	0.252	28/162 (17.3)	151/566 (26.7)	0.017
Aorta arch	336/728 (46.2)	62/149 (41.6)	274/579 (47.3)	0.212	61/162 (37.7)	275/566 (48.6)	0.014
Thoracic descending aorta	319/610 (52.3)	60/128 (46.9)	259/482 (53.7)	0.167	73/136 (53.7)	246/474 (51.9)	0.714
Abdominal aorta	402/718 (56.0)	97/166 (58.4)	305/552 (55.3)	0.469	117/171 (68.4)	285/547 (52.1)	<0.001
Innominate artery	332/775 (42.8)	49/160 (30.6)	283/615 (46.0)	<0.001	59/165 (35.8)	273/610 (44.8)	0.038
Carotid artery	589/775 (76.0)	108/160 (67.5)	481/615 (78.2)	0.005	115/165 (69.7)	474/610 (77.1)	0.033
Subclavian artery	571/775 (73.7)	91/160 (56.9)	480/615 (78.0)	<0.001	114/165 (69.1)	457/610 (74.9)	0.132
Celiac trunk	184/722 (25.5)	35/166 (21.1)	149/556 (26.8)	0.138	48/171 (28.1)	136/551 (24.7)	0.374
Superior mesenteric artery	228/722 (31.6)	51/166 (30.7)	177/556 (31.8)	0.787	71/171 (41.5)	157/551 (28.5)	0.001
Inferior mesenteric artery	28/722 (3.9)	10/166 (6.0)	18/556 (3.2)	0.103	9/171 (5.3)	19/551 (3.4)	0.283
Renal artery	406/727 (55.8)	101/161 (62.7)	305/566 (53.9)	0.046	118/171 (69.0)	288/556 (51.8)	<0.001
Iliac artery	95/710 (13.4)	40/162 (24.7)	55/548 (10.0)	<0.001	18/158 (11.4)	77/552 (13.9)	0.405
Pulmonary artery	124/181 (68.5)	24/33 (72.2)	100/148 (67.6)	0.564	19/29 (65.5)	105/152 (69.1)	0.705
Coronary artery	64/183 (35.0)	11/38(28.9)	53/145(36.6)	0.382	17/47(36.2)	47/136(34.6)	0.842

### 3.3. Angiographic classification between the sexes and age groups of onset

Table 2 lists the distribution of the Numano types by sex and the age group of onset. Overall, type V was the most common pattern irrespective of sex or the age at onset in the TAK patients. In addition to type V, Numano type I (21.0%) was more common in the female patients (*P *= 0.001), while type IV (23.1%) was more likely to occur in the male patients (*P *< 0.001). Compared with the patients with either type I or type II, those with type III, type IV and type V had a relatively lower age peak of onset. The proportions of type I (*P < *0.001) and type IIa (*P *< 0.05) were higher in the adult-onset group than in the childhood-onset group, while the proportions of type IV and type V were higher in the childhood-onset group (*P *< 0.05).

**Table 2:** Sex and age at onset among the different angiographic classifications in TAK patients [*n* (%)].

Numano type	Sex			Age at onset (year)			
	Male (*n* = 182)	Female (*n* = 670)	*P*	Age peak (y)	Age group of onset		
					<18 (*n* = 183)	≥18 (*n* = 669)	*P*
I	19 (10.4)	141 (21.0)	0.001	20–29	18 (9.8)	142 (21.2)	<0.001
IIa	11 (6.0)	30 (4.5)	0.381	25–29	3 (1.6)	38 (5.7)	0.024
IIb	12 (6.6)	57 (8.5)	0.401	25–29	9 (4.9)	60 (9.0)	0.075
III	11 (6.0)	19 (2.8)	0.037	15–19	9 (4.9)	21 (3.1)	0.247
IV	42 (23.1)	69 (10.3)	<0.001	15–19	35 (19.1)	76 (11.4)	0.006
V	87 (47.8)	354 (52.8)	0.228	20–24	109 (59.6)	332(49.6)	0.017

### 3.4. Complications and comparison between the sexes and age groups of onset

The distribution of complications among the sexes and the two age groups of onset in the TAK patients are summarized in Table 3. In the patients with pulmonary hypertension, most of cases (93/103) were evaluated by transthoracic echocardiography and 10 cases were accessed by right heart catheterization. Among all the complications, hypertension was the most prevalent in our TAK cohort irrespective of sex or age at onset. Compared with the female patients, the male patients were more likely to have systemic hypertension (*P *< 0.001), renal dysfunction (*P = *0.040), and aortic aneurysm (*P *= 0.008). However, the female patients had a slightly higher prevalence of pulmonary hypertension than the male patients (*P *= 0.040). The childhood-onset TAK patients had a higher prevalence of systemic hypertension than the adult-onset group (*P *= 0.033).

**Table 3:** Sex, age groups of onset and complications in TAK patients [*n* (%)].

Complications	Total (*n* = 852)	Sex			Age at onset (year)		
		Male (*n* = 182)	Female (*n* = 670)	*P*	<18 (*n* = 183)	≥18 (*n* = 669)	*P*
Systemic hypertension	397 (46.6)	113 (62.1)	284 (42.4)	<0.001	98 (53.6)	299 (44.7)	0.033
Ischemic stroke	89 (10.4)	20 (11.0)	69 (10.3)	0.787	20 (10.9)	69 (10.3)	0.810
Transient cerebral ischemia	37 (4.3)	7 (3.8)	30 (4.5)	0.711	8 (4.4)	29 (4.3)	0.983
Heart ischemia disease	51 (6.0)	12 (6.6)	39 (5.8)	0.697	15 (8.2)	36 (5.4)	0.155
Cardiac dysfunction	119 (14.0)	23 (12.6)	96 (14.3)	0.559	29 (15.8)	90 (13.5)	0.408
Valvular abnormalities	285 (33.5)	68 (37.4)	217 (32.4)	0.207	59 (32.2)	226 (33.8)	0.695
Renal dysfunction	75 (8.8)	23 (12.6)	52 (7.8)	0.040	16 (8.7)	59 (8.8)	0.974
Contracted kidney	60 (7.0)	17 (9.3)	43 (6.4)	0.172	18 (9.8)	42 (6.3)	0.096
Pulmonary hypertension	103 (12.1)	14 (7.7)	89 (13.3)	0.040	18 (9.8)	85 (12.7)	0.291
Aortic aneurysm	39 (4.6)	15 (8.2)	24 (3.6)	0.008	7 (3.8)	32 (4.8)	0.583
Tuberculosis	115 (13.5)	30 (16.5)	85 (12.7)	0.184	23 (12.6)	92 (13.8)	0.678
Infectious diseases of respiratory system	79(9.3)	17(9.3)	62(9.3)	0.971	17(9.3)	62(9.3)	0.993

### 3.5. Association between the angiographic classifications and complications

**Figure 3:**
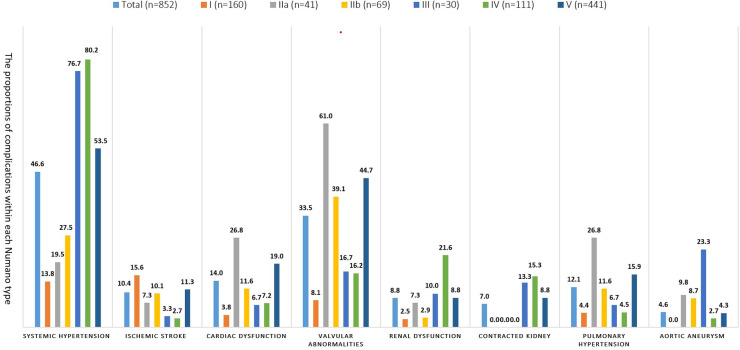
The prevalence of complications within each Numano type in Chinese TA patients. Legend: This figure shows the prevalence of the eight complications that were significantly different among the six Numano angiographic patterns. The horizontal axis represents the types of complications, and the vertical axis represents the prevalence of complications. The columns with different colors indicate the group of the total sample and the different subgroups of the Numano angiographic patterns.

Systemic hypertension (46.6%) and valvular abnormalities (33.5%) were the top two most common complications in the TAK patients. The prevalence of systemic hypertension, ischemic stroke, cardiac dysfunction, valvular abnormalities, renal dysfunction, contracted kidney, pulmonary hypertension, and aortic aneurysm was found to be significantly different among the angiographic patterns (*P *< 0.001 for all). The detailed prevalence of the above complications within each Numano angiographic pattern is shown in Figure 3. The patients with type I showed a tendency to have ischemic stroke (15.6%). After adjusting for sex and age at onset, the patients with type II were associated with an increased risk of cardiac dysfunction (II vs. I: OR = 5.42; II vs. IV: OR = 2.63) and pulmonary hypertension (II vs. I: OR = 4.78; II vs. IV: OR = 3.95) compared with those with types I and IV. Valvular abnormalities (61.0%) were observed to be most prevalent in the TAK patients with type IIa. The patients with Type III were associated with a higher risk of AA (23.3%) than the patients with types IV (OR = 11.00) and V (OR = 5.98) and had an increased risk of systemic hypertension than those with types I (OR = 18.98) and II (OR = 9.86). The patients with type IV were more commonly complicated with systemic hypertension (IV vs. I: OR = 24.20,; IV vs. II: OR = 12.57; IV vs. V: OR = 3.30) and renal dysfunction (IV vs. I: OR = 11.45; IV vs. II: OR = 6.49; IV vs. V:OR = 2.87) than the patients with types I, II and V. *P *< 0.05 in all of the above comparisons. The patients with type V had more diverse complications and were demonstrated to have a higher risk of valvular abnormalities and pulmonary hypertension than those with types I and IV and were also more likely to develop systemic hypertension, renal and cardiac dysfunction than the patients with type I in our cohort. The associations between the different Numano types and complications adjusted by age at onset and gender using a binary logistic regression model are detailed in Table 4.

**Table 4:** Association between the different numano types and complications among Chinese TAK patients after adjusting for age at onset and gender.

Complications	Numano types	OR (95% CI)			
Hypertension	I	Ref.			
	II	1.93 (1.033.62)*	Ref.		
	III	18.98 (7.22–49.88)*	9.86 (3.78–25.71)*	Ref.	
	IV	24.20 (12.53–46.78)*	12.57 (6.57–24.03)*	1.28 (0.48–3.38)	Ref.
	V	7.34 (4.48–12.01)*	3.81 (2.36–6.17)*	0.39 (0.16–0.93)*	0.30 (0.18–0.50)*
Ischemic stroke	I	Ref.			
	II	0.52 (0.24–1.13)	Ref.		
	III	0.18 (0.02–1.36)	0.34 (0.04–2.78)	Ref.	
	IV	0.15 (0.04–0.52) *	0.29 (0.08–1.09)	0.85 (0.09–8.51)	Ref.
	V	0.71 (0.42–1.20)	1.37 (0.67–2.81)	4.02 (0.53–30.29)	4.73 (1.44–15.54)*
Cardiac dysfunction	I	Ref.			
	II	5.42 (2.09–14.09)*	Ref.		
	III	1.89 (0.36–9.88)	0.35 (0.08–1.59)	Ref.	
	IV	2.05 (0.69–6.15)	0.38 (0.16–0.91)*	1.09 (0.22–5.41)	Ref.
	V	6.09 (2.60–14.27)*	1.12 (0.65–1.95)	3.22 (0.75–13.83)	2.97 (1.38–6.34)*
Valvular abnormalities	I	Ref.			
	II	9.90 (5.01–19.56)*	Ref.		
	III	2.14 (0.70–6.55)	0.22 (0.08–0.61)*	Ref.	
	IV	2.12 (0.98–4.57)	0.21 (0.11–0.41)*	0.99 (0.33–2.95)	Ref.
	V	9.25 (5.08–16.86)*	0.94 (0.61–1.43)	4.33 (1.62–11.59)*	4.37 (2.53–7.54)*
Renal dysfunction	I	Ref.			
	II	1.76 (0.46–6.75)	Ref.		
	III	4.14 (0.87–19.78)	2.35 (0.52–10.55)	Ref.	
	IV	11.45 (3.78–34.62)*	6.49 (2.34–17.98)*	2.77 (0.76–10.06)	Ref.
	V	3.99 (1.40–11.39)*	2.26 (0.87–5.91)	0.96 (0.28–3.37)	0.35 (0.20–0.62)*
Pulmonary hypertension	I	Ref.			
	II	4.78 (1.93–11.84)*	Ref.		
	III	1.78 (0.35–9.06)	0.37 (0.08–1.71)	Ref.	
	IV	1.21 (0.37–3.93)	0.25 (0.09–0.71)*	0.68 (0.12–3.70)	Ref.
	V	4.38 (1.96–9.79)*	0.76 (0.52–1.61)	2.47 (0.57–10.65)	3.64 (1.43–9.29)*
Aortic aneurysm^a^	II	Ref.			
	III	2.75 (0.93–8.14)	Ref.		
	IV	0.25 (0.06–0.94)*	0.09 (0.02–0.37)*	Ref.	
	V	0.46(0.21–1.03)	0.17(0.06–0.44)*	1.88(0.54–6.5)	

Ref. refers to the reference group set for multiple comparisons. OR values indicate each comparison result between the group with a specific Numano type and the reference group was obtained from the binary logistic regression mode adjusted by the age at onset and gender of the TAK patients.

^a^ The results did not include a comparison with type I due to no case with aortic aneurysm was identified in type I patients. Since the sample sizes of the patients with type IIa and type IIb were small, we merged the two groups into one group for further analysis.

* *P *< 0.05.

## 4. Discussion

In our study, we identified the demographic characteristics, vessel involvement, Numano types, complications and associations among these data in Chinese patients with TAK, which is noteworthy, as these retrospective data are sourced from a large single tertiary referral medical center in China. There are several large studies that have described the broad clinical features of TAK patients in China (20, 27–29), but the larger sample enrolled in our study enabled us carry out meaningful comparisons between subgroups of sex, age of onset regarding vessel involvement, angiographic patterns and complications. Furthermore, thanks to the large series of patients, we first attempt to evaluate the risk of complications in the subgroups of different Numano angiographic types in China.

Research focusing on TAK demographic characteristics demonstrates that the female-to-male ratio in China is similar to that in Thailand, higher than that in India, but significantly lower than that in Japan, Italy, Turkey, Mexico and South Korea (9). Regional differences in the incidences of TAK in female and male patients may be affected by genetic factors, dietary habits and environmental conditions (9). The F:M ratio was lower in our series than in most of the results reported from European and South American countries (4, 5, 30, 31). This finding further verified these graphical differences in the sex ratio in TAK patients. We did not find differences in the sex ratio between the childhood-onset group and the adult-onset group of TAK. However, a lower female predominance has been found previously in some studies of pediatric-onset TAK compared to adult-onset TAK cohorts (8, 27, 32, 33). We supposed that the different results were partially because of inconsistencies between the age at onset and the age at diagnosis applied in different studies; on the other hand, the different results may be due to the features of TAK, including its insidious onset and rare occurrence, which would make it difficult to obtain a completely accurate age at onset from TAK patients. Genetic factors such as HLA-B* 52 have been reported to be particularly associated with an earlier disease onset (34). The possible underlying causal mechanisms of sex and age on onset still need to be further investigated.

The angiographic pattern of TAK patients revealed variations in different series, and either type I or type V was the most frequent (20, 22, 30, 23, 24, 35–37). Numano type I is reported to be predominant in TAK patients from Iran (22), Norway (38) and Japan (2), whereas Numano type V is the predominant type reported in China (20), South Korea (24), Turkey (30), and Thailand (39). We observed the same predominance of Numano type V in our cohort. These findings may be to some extent attributed to referral bias that resulted from more severe TAK patients with a broader extent of vessel involvement being recruited to the study hospital. In addition, the disease progression during the long span of our study was also responsible for the higher proportion of type V patients.

Similar to previous studies (2, 8, 9, 29 ), our study strongly indicates that sex could play an important role in the pattern of vascular lesions and consequently in the corresponding complications in Chinese TAK patients. We found that female patients more commonly had involvement of the branches of the aortic arch, whereas in men, the iliac arteries and renal arteries were more frequently affected. Similar patterns of vascular lesions in males (4, 5, 9) and females (3, 5, 9) have also been reported by several other studies. Our findings demonstrated that women have a higher involvement of the supradiaphragmatic vessels; in contrast, men were more likely to have involvement of the abdominal vessels. In accordance with the results reported from Korea (5), we found that males had a higher incidence of type IV than females, while females showed a significantly higher incidence of type I than males. This could be clearly illustrated by the significantly higher proportion of females that had involvement of aortic arch branches and the tendency for males in our cohort to have involvement of the renal arteries. In addition, it is well known that sex could have a strong influence on the prognosis of several rheumatic diseases. For example, men tend to have a worse prognosis from systemic lupus erythematosus (40). A large cohort study of TAK from Japan showed that males had a higher prevalence of extensive aortic involvement (2) and another study from China demonstrated that male patients were more prone to present with multivessel involvement (29). Other studies with relatively large cohorts also showed that aortic aneurysm (6, 7, 41), renal dysfunction and systemic hypertension (3) developed more frequently in male patients and were regarded as the main mortality predictors in TAK patients (31). In line with those studies, our results showed the same strong association between the presence of the above three complications and male sex, thereby correlating male sex to a poor prognostic factor. This could also be further exacerbated by a poor lifestyle, such as tobacco smoking, which has been demonstrated to be a possible contributor in male patients (41–43).

It has been reported that the distribution of vascular involvement is different between childhood-onset and adult-onset TAK patients (8, 33, 44). Childhood-onset TAK patients presented with more severe inflammation and more widespread vessel lesions (44). According to the Numano classification, a similar tendency was recognized in our study. We observed that the proportion of patients with type V was higher in the childhood-onset patients than in the adult-onset group, implying that more extensive vascular lesions more frequently occur in childhood-onset patients. In other pediatric cohorts, children had significantly more aortic and renal artery involvement than adult-onset patients (8, 33), and another study from China also revealed that renal arteries and abdominal aorta are the major sites of vascular lesions in childhood-onset TAK patients (45). These findings are also largely validated by our study. Similar to other studies (2, 23, 24, 31), systemic hypertension was the most common complication in our TAK series irrespective of sex and age. We found that the childhood-onset group had a higher prevalence of systemic hypertension than the adult-onset group, which could be illustrated by the features of the vascular lesions in this subgroup. Therefore, when systemic hypertension is diagnosed in children in routine clinical practice, TAK should be highly suspected.

It is logical that the angiographic pattern is closely associated with the corresponding complications in TAK patients; however, very few studies have shown their association in a relatively large sample of TAK patients. We found that 10.4% of our TAK patients had ischemic strokes, with the highest prevalence in the patients with type I, which was similar to the pooled prevalence of stroke (8.9%) reported by a meta-analysis for ischemic complications in TAK patients (46). A national population survey of 480,687 adults from China revealed that the age-standardized prevalence of stroke was 1,114.8/100,000 people, of which 77.8% of the patients had ischemic stroke (47), suggesting up to an approximately 11-fold increase in the prevalence of ischemic stroke in TAK patients based on our findings. Variances in the occurrence of ischemic stroke have been noted in studies with different ethnic groups, showing that patients originating from North Africa had a more frequent occurrence of ischemic stroke than white patients (25.0% vs. 5.13%) (48). Based on our study and a study from Japan, it is inferred that in TAK patients, the occurrence of ischemic stroke in Asians was possibly higher than that in white patients and lower than that in patients from North Africa. We reported a higher prevalence of 10.9% in childhood-onset Chinese TAK patients compared with the pooled prevalence of 7.7% that was reported in the same subgroup by another study (46). These data suggest that ischemic stroke is not a rare complication either in childhood-onset or adulthood-onset TAK patients.

In addition to ischemic stroke, cardiopulmonary abnormalities are also not rare in TAK patients (2, 16, 49). The reported prevalence of pulmonary hypertension varied from 2.4% to 40.7% (13, 49–52), and the differences among the studies might result from both the method used to detect the pulmonary pressure measurement and the differences of these cohorts. Similar with 13.0% reported in a large cohort of 216 Chinese TAK patients (51), pulmonary hypertension was documented in 12.1% of our cohort. However, this prevalence might be overestimated in the two studies due to their evaluation upon echocardiography rather than right heart catheterization in the majority of the patients. A previous study from China (20) reported that multiple cardiac abnormalities were more frequent in patients with type V, suggesting that these complications might be more common in TAK patients with longer disease duration. However, in our series, in addition to type V, we also observed the highest prevalence of valvular abnormalities, cardiac dysfunction and pulmonary hypertension in patients with type IIa. This could be due to the discrepancy in data collection or the inclusion criteria in different studies and could be also attributed to the possible underestimation of mild cardiopulmonary complications, which doctors may fail to document in the discharge abstract and may not be completely identified in our study because this study only included the ICD-10 codes noted during discharge. Nevertheless, it is well known that valvular abnormalities of aortic regurgitation are often caused by proximal aortic root dilation, which could also result in subsequent pulmonary hypertension and cardiac insufficiency. In addition, elevated left ventricular or atrial filling pressure due to ascending aortic lesions contributes to the enlargement of cardiac chambers, especially the left ventricle, which indirectly leads to cardiac dysfunction. Thus, it is rational to assume that vessel lesions involved in type II could generally explain the mechanism resulting in the corresponding complications. Before irreversible pathophysiologic changes occur, it is strongly recommended to have Doppler echocardiography performed in patients with type II or type V when the diagnosis of TAK is established.

We found 4.6% of our TAK patients had AA, and AA incidences of 4.1%–16.6% were reported in previous studies (2, 7, 20, 41). The variance of incidences might be largely due to the selection bias among these studies. Several studies have shown that ascending aortic aneurysms and abdominal aneurysm are the most common types of AA in TAK patients (7, 41, 53). Angiographic type V was reported to be the most frequent pattern in Chinese TAK patients with AA (7). However, in our cohort, we found that AA occurred most frequently in patients with type III. This may be partially because patients with type III had lesions that involved the whole extent of the descending aorta, including most of the high-risk anatomical sites of AA, while in type V, the vessel lesions could have various patterns that may not always cover the high-risk sites of AA. It is highly suggested that more attention should be given to patients with all extents of descending aorta involvement to better evaluate and prevent AA in TAK patients. Unlike AA, we observed the same often-reported association of renal artery involvement and systemic hypertension as reported by other series (49). Patients with type III and type IV showed an extremely higher risk of systemic hypertension than those with type I, type II and type V, which demonstrates that the involvement of the renal artery and abdominal aorta could be the main mechanism leading to renovascular hypertension in TAK patients.

Our results should be interpreted while taking some limitations into account. First, this study was retrospectively performed in a single medical center; thus, more representative population-based studies on TAK should be conducted to explore the epidemiological features of TAK patients in China and the findings may not be extrapolated to other settings. Secondly, as a national referral center, the data from PUMCH may not include patients with no symptoms or patients with mild symptoms who were treated in local hospitals. Therefore, possible referral bias should be considered in our study setting. Finally, this study must be interpreted in light of limitations inherent to its retrospective design. Since hospital inpatient data on angiographic findings and complications tend to be recorded with variable diligence and completeness, a prospective study is needed to investigate the inter-relation between demographic subgroups, angiographic subgroups and complications in Chinese TAK patients.

In conclusion, our study showed that there were age-specific and gender-specific differences between the different vascular lesions, angiographic classifications and complications in a large sample of Chinese TAK patients. Our findings demonstrated that women have a higher involvement of the supradiaphragmatic vessels; in contrast, men were more likely to have involvement of the abdominal vessels. Male patients had a higher prevalence of systemic hypertension, renal dysfunction and aortic aneurysm, while female patients had a slightly higher likelihood of pulmonary hypertension than males. The childhood-onset group more frequently had involvement of the subdiaphragmatic arteries, also revealed more extensive vascular lesions and had a higher prevalence of systemic hypertension. The Numano angiographic patterns were closely associated with the risk of complications; specifically, patients with Numano type II, type IV and type III had a remarkable risk of developing cardiopulmonary abnormalities, systemic hypertension and AA, respectively. These findings suggest male and childhood-onset TAK patients should be paid more attention to in terms of the disease diagnosis and assessment. Comprehensive evaluation of vascular involvement is quite necessary for TAK when diagnosed and need routine monitoring to improve prognosis.

## Data Availability

The raw data supporting the conclusions of this article will be made available by the authors, without undue reservation.

## Supplementary material

SUPPLEMENTARY FIGURE S1

Distribution of age at onset by sexes in Chinese TAK patients. This figure shows the distribution of age at onset for male and female TAK patients in our study. The red bars indicate the female group and the blue ones represent the male group.
