# Supplementary figures and images for: Age, sex and angiographic type-related phenotypic differences in inpatients with Takayasu arteritis: A 13-year retrospective study at a national referral center in China
Source: Front Cardiovasc Med. 2023 Mar 16;10:1099144. doi: 10.3389/fcvm.2023.1099144 (PMC10062600; doi:10.3389/fcvm.2023.1099144)

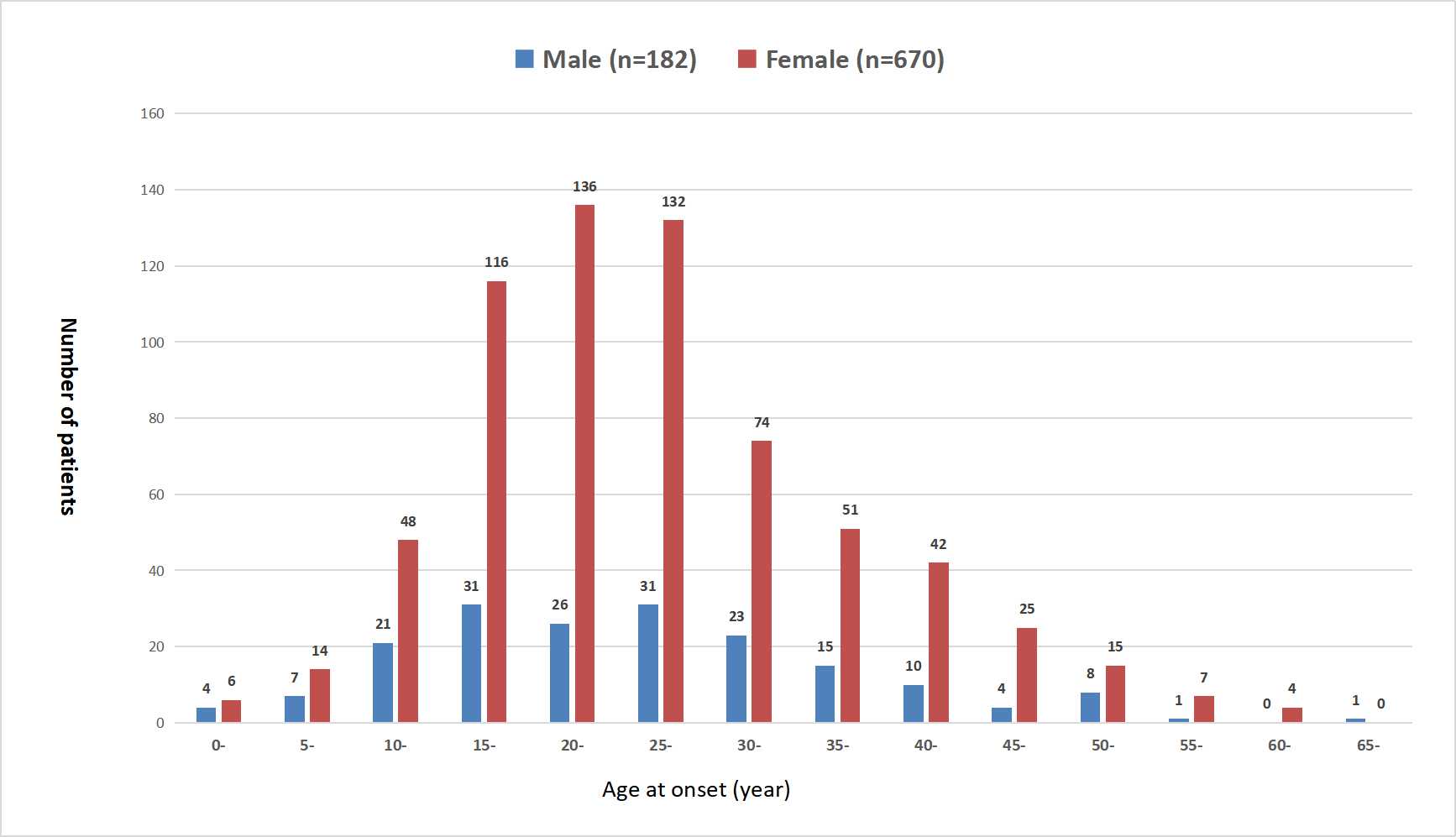

Supplement: Supplementary file 1 [file Image1.jpeg]
